# Factors Contributing to Diagnosis and Prognosis in Sinonasal Malignancies

**DOI:** 10.1155/2022/4406838

**Published:** 2022-09-29

**Authors:** Wenjing Li, Jianqiang You, Haixiang Xue, Changjiang Chao

**Affiliations:** E.N.T. Department, The Third Affiliated Hospital of Soochow University, Changzhou, Jiangsu 213000, China

## Abstract

**Objectives:**

This study was intended to explore and analyze the factors which affect the survival and prognosis of patients with malignant tumors of nasal cavity and sinus.

**Methods:**

Retrospective analysis was performed on the clinical data of 39 cases of malignant tumors of nasal cavity and sinus that met the requirements of the study. A follow-up study was performed on the patients for more than 36 months. Survival analysis was conducted via the Kaplan-Meier method and log-rank test. Cox regression model was used for multivariate analysis.

**Results:**

Gender, pathological type, treatment plan, clinical stage, and survival time of patients were different. Clinical stage was substantially related to the survival of patients (*P* < 0.05), which was an independent factor affecting prognosis.

**Conclusions:**

Early detection and comprehensive treatment of sinonasal malignancies can improve the prognosis and prolong the survival time of patients.

## 1. Introduction

Malignant tumors of nasal cavity and sinus account for 0.2 to 0.5% of all human cancers and 3% of head and neck cancers in Western countries [1]. Nearly 95% of maxillary sinus carcinomas occur in patients over 40 years old; however, sarcomas are more common in young adults and children [2]. Squamous cell carcinoma is common and often occurs in the maxillary sinus. Adenocarcinoma and adenoid cystic carcinoma are less frequent than squamous cell carcinoma and often occur in the ethmoid sinus [3]. Most malignant tumors of the nasal cavity and sinus start with atypical symptoms in early stage; moreover, it is difficult to make early diagnosis because of the hidden and complicated anatomy of nasal cavity and sinus [4]. The aim of this retrospective review was to explore the relevant factors affecting the prognosis in malignant tumors of the nasal cavity and sinus and then guide the clinical treatment.

## 2. Materials and Methods

### 2.1. Clinical Data

Retrospective analysis was performed on the clinical data of 39 patients admitted to the hospital with “malignant tumors of the nasal cavity and sinus” from February 2009 to February 2019. All patients were followed up, for periods ranging between 36 to 80 months, with an interval of 3 months between each follow-up, by telephone and letter. The patients' referral statuses were obtained, so as to evaluate the current condition and tumors status of the patients. 5 patients were lost, with a follow-up rate of 87.2%. The study was approved by the ethics committee of the Third Affiliated Hospital of Soochow University, and written informed consent was obtained from all patients.

### 2.2. Inclusion and Exclusion Criteria

Patients with benign tumors, such as inverted papilloma, and with palate or skin primary tumors with secondary invasion of the sinuses and nose were excluded. Patients with nasal vestibule primary tumors also were excluded due to these tumors that probably are related more to skin primary tumors than to nasal carcinoma. Only patients who were treated primarily and follow-up between 36 to 80 months were included.

### 2.3. Statistical Analysis

The Kaplan-Meier method and the log-rank test were utilized to analyze the survival rate of patients, while the Cox regression model was used for the multivariate analysis. *P* < 0.05 was considered to indicate a statistically significant difference.

## 3. Results

### 3.1. Clinical Characteristics

The clinical characteristics of 39 patients are as shown in Table 1. Squamous cell carcinoma was the most common pathological type. Surgical resection (*n* = 12) and surgery combined with other auxiliary treatments (*n* = 18) were the major therapeutic methods. Stage IV (*n* = 17) was the most common clinical stage.

### 3.2. Surgical Resection Pathologic Data

The majority of patients did not have evidence of perineural invasion, lymphatic invasion, or angioinvasion (Table 2).

### 3.3. Survival and Prognosis Analysis

**Figure 1:**
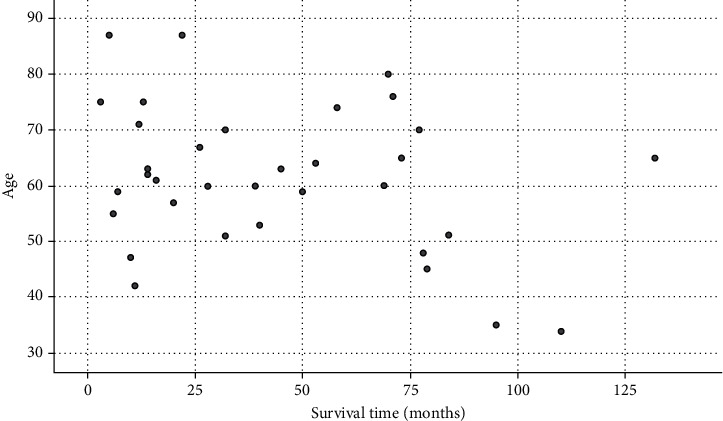
The scatter diagram of patients' age and survival time.

**Figure 2:**
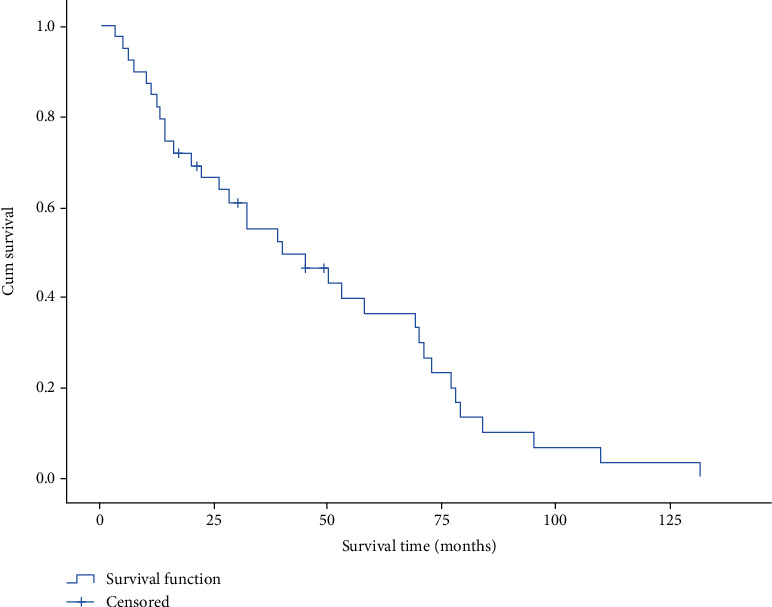
Overall survival curve.

**Figure 3:**
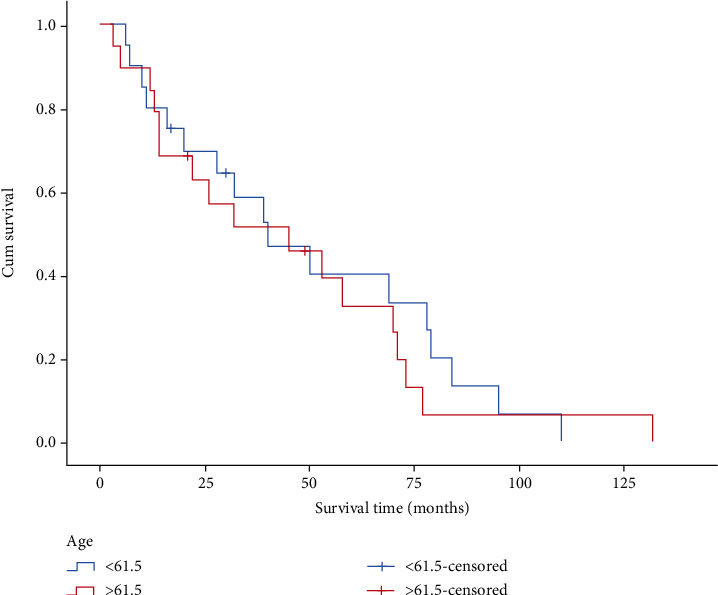
Survival curves of different age groups.

The survival time of 39 patients varies from 3 to 132 months.  Figure 1 is the scatter diagram of patients' age and survival time. The overall mean survival time was 47.3 months. The median survival time was 40 months. The survival curve is shown in Figure 2. The longer the survival time, the lower the survival rate. The oldest patient was 87 years old; the youngest patient was 34 years old; the average age was 61.3; the median age was 61.5. The median survival time of patients younger than 61.5 years was 49.3 months, while the median survival time of patients older than 61.5 years was 45.1 months. As exhibited in Figure 3, there was no significant difference between the median survival time of patients younger and older than 61.5 years old after the removal of discrete values (*P* = 0.63).

**Figure 4:**
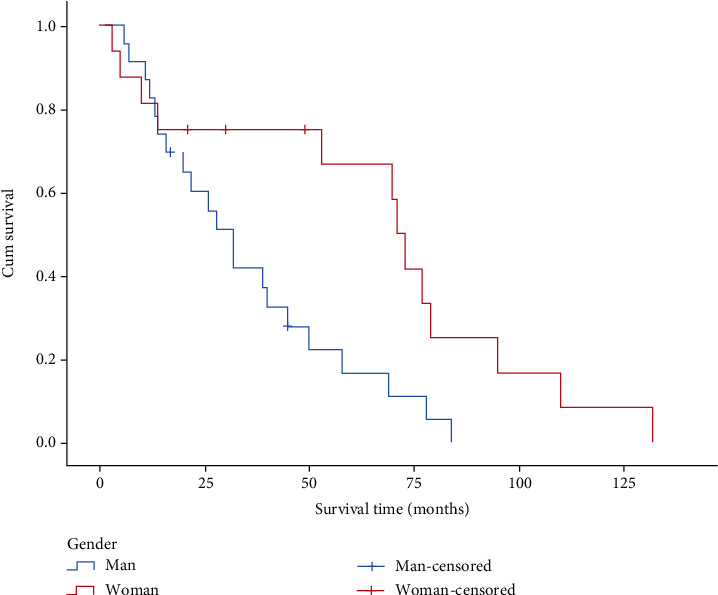
Survival curves of different sex groups.

**Figure 5:**
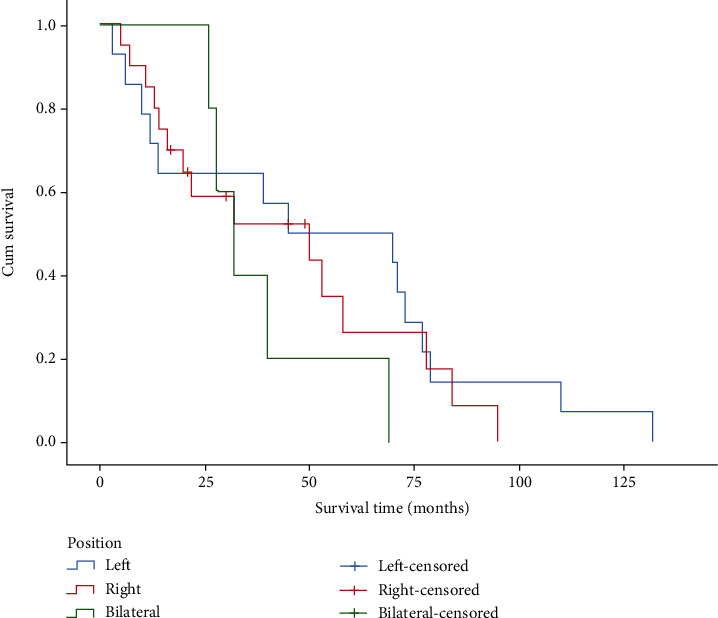
Survival curve of different parts groups.

There were 23 male patients and 16 female patients. The median survival time was 27 months for male patients and 61.5 months for female patients. As indicated in Figure 4, there was a significant difference between the survival time of male and female patients after the removal of discrete values (*P* = 0.01). Among 39 cases, there were 5 cases of bilateral nasal involvement, 20 cases of right nasal involvement, and 14 cases of left nasal involvement. As shown in Figure 5, the difference in survival time of patients with different locations of sinonasal tumors was statistically significant (*P* = 0.477).

**Figure 6:**
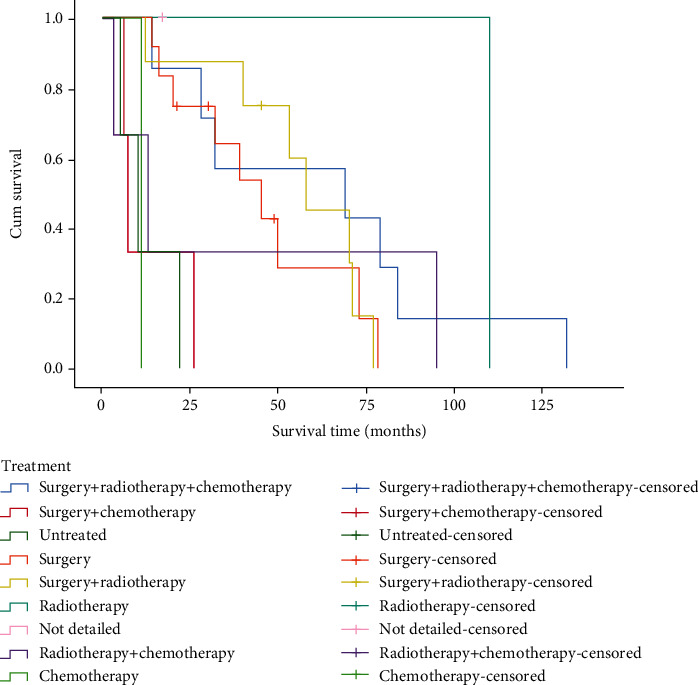
Survival curves of different treatment groups.

**Figure 7:**
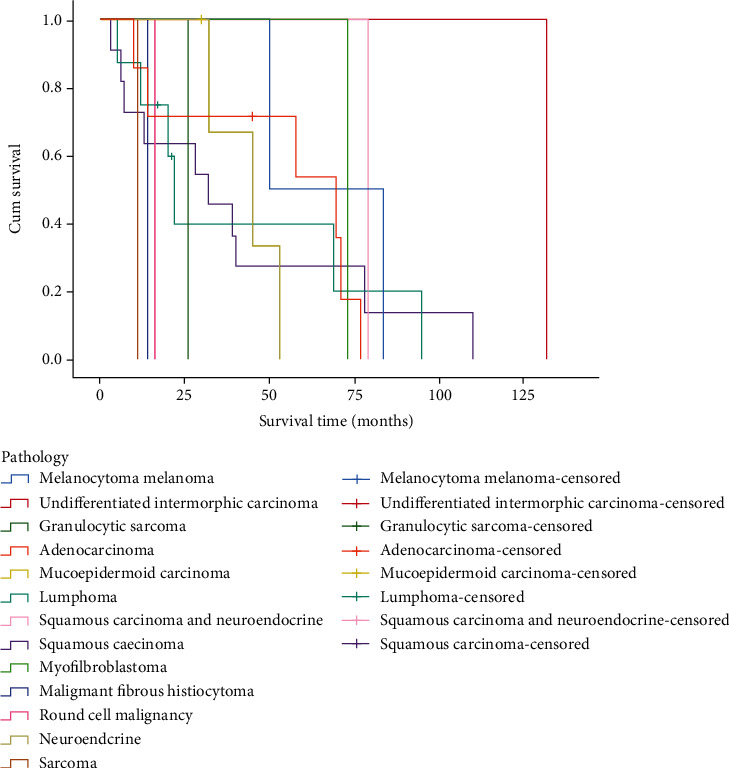
Survival curve of different pathological groups.

**Figure 8:**
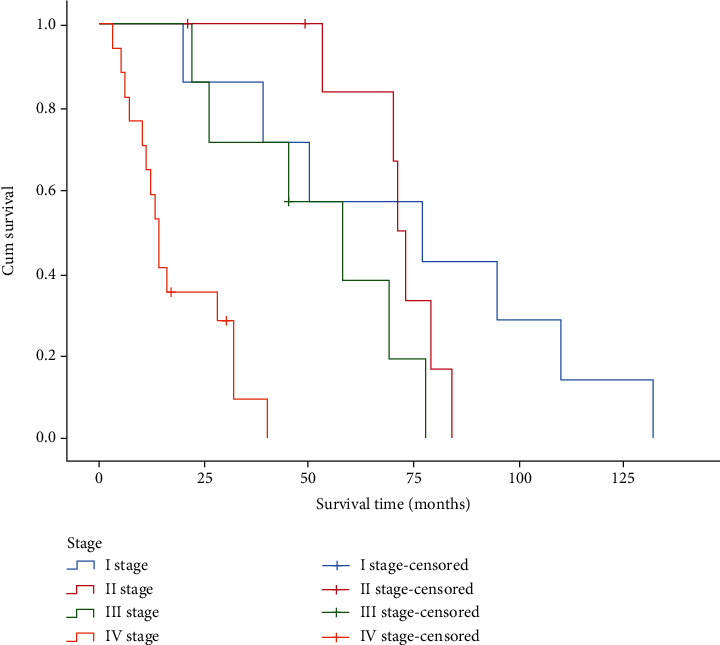
Survival curves of different clinical stage groups.

Among 39 patients, 7 patients received surgery and adjuvant radiotherapy and chemotherapy; 3 patients underwent surgery and adjuvant chemotherapy; 3 patients received no treatment; 12 patients only received surgical treatment; 8 patients received surgical treatment and adjuvant radiotherapy; 1 patient received radiotherapy only; 1 patient received unknown treatment; 3 patients received radiotherapy and chemotherapy; and 1 patient received chemotherapy only. As illustrated in Figure 6, there was a significant difference in the survival time of patients receiving different treatments (*P* < 0.001). The pathological types and survival time of 39 patients could be seen in Figure 7. The log-rank test showed that the survival difference was statistically significant (*P* = 0.02). Clinical stages were determined based on the TNM staging criteria of the 7th edition American Joint Committee on Cancer (AJCC) in 2010. There were 7 patients at Stage I, 8 patients at Stage II, 7 patients at Stage III, and 17 patients at Stage IV.  Figure 8 indicates a statistically significant difference in survival time of patients with sinonasal tumors at different stages.

### 3.4. Evaluation of Prognostic Factors

**Figure 9:**
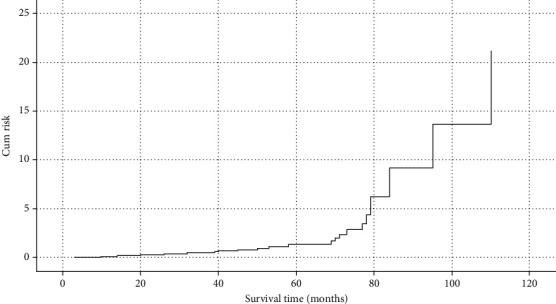
The risk function of covariates in the Cox regression analysis.

The Cox proportional risk regression model was established to analyze the prognostic factors which included the patient's age, gender, pathological type, treatment plan, and clinical stage. The results showed that clinical stage (Stage IV hazard ratio (HR),  2.93; 95% confidence interval (CI), 1.68 to 5.12; *P* < 0.001) was an independent factor affecting the prognosis (Figure 9).

## 4. Discussion

Sinonasal tumors are mainly derived from the epithelium and mesenchymal cells of the nasal cavity and sinus. Based on the origin of tumors, sinonasal tumors can be divided into diversified pathological types [5]. Most tumors originate from epithelial tissue. In this study, the pathological types include melanoma, undifferentiated anaplastic carcinoma, granulocytic sarcoma, adenocarcinoma, mucoepidermoid carcinoma, lymphoma, squamous cell carcinoma, myofibroblastoma, malignant fibrohistiocytoma, round cell malignancy, neuroendocrine carcinoma, sarcoma, and squamous cell carcinoma with neuroendocrine carcinoma. Among them, squamous cell carcinoma and adenocarcinoma were more common. The etiologies are related to the Epstein-Barr virus or carcinogenic factors in the environment, such as smoking, dust, and exposure to chemical adhesives [6]. In our study, there were 16 female patients and 23 male patients, with a median survival time of 27 months for male patients and 61.5 months for female patients. The difference was statistically significant. The morbidity was higher in men than in women. The survival time of male patients is shorter than that of female patients probably because most men smoke. Studies have shown that VCA-IgA exhibits the highest positive rate among three antibodies of the Epstein-Barr virus (VCA-IgA, EA-IgA, and Rta-IgG) and was higher in men than in women [7]. The positive rate of three kinds of EBV antibody increased with age in different age groups. The positive rates of Ea-IgA (*P* < 0.001) and Rta-IgG (*P* = 0.0038) were significantly different in different age groups [8].

Sinonasal tumors exhibit no typical clinical symptoms in early stage [9]. Major symptoms include nasal obstruction, running nose, and epistaxis. In advanced stages, symptoms such as headache, decreased sense of smell, facial swelling and numbness, inferior vision, and prominent eyeball may appear [10]. In this study, nasal obstruction (49.2%) and epistaxis (28.8%) were major clinical manifestations in early stage. The primary locations of the sinonasal tumors included nasal cavity (30.4%), maxillary sinus (26.6%), ethmoid sinus (18.9%), sphenoid sinus (11.4%), nasal septum (5.1%), frontal sinus (2.5%), turbinate (2.5%), and nasal root (1.3%).

Early diagnosis is considered a key factor to improve outcomes in cancer therapy. Nevertheless, limited anatomic access makes early diagnosis difficult. The air-filled spaces allow asymptomatic growth till invasion into adjacent structures [11]. In addition, nasal discharge, obstruction, or epistaxis may also occur if the medial wall of the sinus is invaded [12]. When epistaxis, headache, or visual loss occurs, the tumor may be advanced.

Imaging examinations such as CT and MRI are currently used as auxiliary examination methods. The squamous cell carcinoma of the nasal cavity and sinus demonstrate complete osteolytic destruction on CT, CT examination of adenoid cystic carcinoma showed that the bone destruction was slowly dilated, and the bone was compressed and thinned in the middle of the tumor, which could be accompanied by bone destruction in the later stage [13]. The diagnostic significance of MRI is to show the internal structure and invasion range of the tumor. MRI examinations of adenoid cystic carcinoma display uneven internal signal with enhancement, neural tube foramen enlargement, and soft tissue invasion. The affected nerve is thickened and strengthened [14]. CT combined with MRI can make a preliminary judgment on benign or malignant tumors and the stage of the tumor. But the final diagnosis is based on pathology.

Sinonasal tumors are usually surgically removed after pathological diagnosis. Then, radiotherapy, chemotherapy, and other auxiliary treatments are applied to prolong the survival time of patients [15]. Previous surgical treatment methods include nasal incision, total maxillary resection, partial maxillary resection, enlarged maxillary resection, and craniofacial resection. The surgical approaches mainly include facial approach, transcranial approach, and craniofacial approach. In clinical practice, the best surgical approach is usually selected according to tumor location and invasion range. However, surgical operations may lead to facial deformity, postoperative craniocerebral complications, and intracranial infection [16], which should be selected with caution in comprehensive clinical practice. At present, the major surgical method for sinonasal tumors is nasal endoscopic surgery which provides a better quality of life for patients than traditional open surgery [17]. For nasal adenoid cystic carcinoma and nasal melanoma, studies have shown that there is no significant difference in survival rate between open surgery and endoscopic surgery, which may be related to high malignance of these tumors [18]. The recurrence rate of other pathology needs to be further studied.

Numerous studies have demonstrated that the tumor stage is closely related to the survival rate of patients [19, 20]. In spite of the small sample size of our study, it was found that patients with early-stage sinonasal tumors had better prognosis than those with sinonasal tumors at more advanced stages. The latter still face poor outcomes after radiotherapy or chemotherapy [21]. Researchers have observed longer 5-year survival in adenocarcinoma cases than in undifferentiated carcinoma cases [22], suggesting pathological type could be an important factor for the prognosis of patients with sinonasal tumors. However, Martínez-Rodríguez et al. found no significant relations between pathological type and survival time of patients [21].

Guntinas-Lichius et al. disclosed that surgery is a preferred therapeutic method for patients with sinonasal tumors at Stage I or Stage II [23]. They also stated that the outcomes of patients with tumors at Stage III or Stage IV were unsatisfied, and notwithstanding multimodality treatment may improve prognosis. So far, no controlled trials have confirmed the efficacy of chemotherapy and radiotherapy in improving the survival rate of patients with frontal sinus tumors; however, these treatments are conducive to local control [24]. Recently, intra-arterial infusion chemotherapy combined with radiotherapy has been performed for patients with locally advanced sinonasal tumors, which results in favorable outcomes [25, 26].

In this study, most of the patients were treated with comprehensive therapy, and the difference in survival was significant in statistics (*P* < 0.001). In addition, domestic studies of nasal cavity and sinus squamous cell carcinoma have pointed out that the treatment of preoperative radiotherapy combined with surgery or surgery combined with postoperative radiotherapy is better than surgery or radiotherapy in the treatment of patients in the middle and late stages.

## Figures and Tables

**Table 1:** Clinical data of 39 patients with malignant tumor of nasal cavity and sinus.

Category	Case	Mean survival time (months)	Median survival time (months)
Pathological type			
Melanocytoma melanoma	2	67	67
Undifferentiated intermorphic carcinoma	1	132	132
Granulocytic sarcoma	1	26	26
Adenocarcinoma	7	49.3	58
Mucoepidermoid carcinoma	1	30	30
Lymphoma	8	32.6	20.5
Squamous carcinoma and neuroendocrine	1	79	79
Squamous carcinoma	11	36.8	32
Myofibroblastoma	1	73	73
Malignant fibrous histiocytoma	1	14	14
Round cell malignancy	1	16	16
Neuroendocrine	3	43.3	45
Sarcoma	1	11	11
Treatment method			
Surgery+radioterapy+chemotherapy	7	62.6	69
Surgery+chemotherapy	3	13	7
Untreated	3	12.3	10
Surgery	12	38.9	35.5
Surgery+radioterapy	8	53.3	55.5
Radiotherapy	1	110	110
Not detailed	1	17	17
Radiotherapy+chemotherapy	3	37	13
Chemotherapy	1	11	11
Clinical stage			
Stage I	7	74.7	77
Stage II	8	71.7	71
Stage III	7	52.3	58
Stage IV	17	18.3	14

**Table 2:** Surgical resection pathologic data.

Characteristic	*n*	%
Lymphatic invasion		
No	38	97.4
Yes	1	2.6
Perineural invasion		
No	37	94.9
Yes	2	5.1
Angioinvasion		
No	39	100
Yes	0	

## Data Availability

The data used to support the findings of this study are included within the article.
